# Label-free cell cycle analysis for high-throughput imaging flow cytometry

**DOI:** 10.1038/ncomms10256

**Published:** 2016-01-07

**Authors:** Thomas Blasi, Holger Hennig, Huw D. Summers, Fabian J. Theis, Joana Cerveira, James O. Patterson, Derek Davies, Andrew Filby, Anne E. Carpenter, Paul Rees

**Affiliations:** 1 Imaging Platform at the Broad Institute of Harvard and MIT, 415 Main St, Cambridge, Massachusetts 02142, USA; 2 Helmholtz Zentrum München—German Research Center for Environmental Health, Institute of Computational Biology, Ingolstädter Landstraße 1, 85764 Neuherberg, Germany; 3 Department of Mathematics, Technische Universität München, Boltzmannstraße 3, 85748 Garching, Germany; 4 College of Engineering, Swansea University, Singleton Park, Swansea SA2 8PP, UK; 5 Flow Cytometry Facility, The Francis Crick Institute, Lincoln's Inn Fields Laboratory, 44 Lincoln's Inn Fields, London WC2A 3LY, UK; 6 Cell Cycle Laboratory, The Francis Crick Institute, 44 Lincoln's Inn Fields, Holborn WC2A 3LY, UK; 7 Newcastle Upon Tyne University, Faculty of Medical Sciences, Bioscience Centre, International Centre for life, Newcastle Upon Tyne NE1 7RU, UK

## Abstract

Imaging flow cytometry combines the high-throughput capabilities of conventional flow cytometry with single-cell imaging. Here we demonstrate label-free prediction of DNA content and quantification of the mitotic cell cycle phases by applying supervised machine learning to morphological features extracted from brightfield and the typically ignored darkfield images of cells from an imaging flow cytometer. This method facilitates non-destructive monitoring of cells avoiding potentially confounding effects of fluorescent stains while maximizing available fluorescence channels. The method is effective in cell cycle analysis for mammalian cells, both fixed and live, and accurately assesses the impact of a cell cycle mitotic phase blocking agent. As the same method is effective in predicting the DNA content of fission yeast, it is likely to have a broad application to other cell types.

## Abstract (web summary)

Imaging flow cytometry enables high-throughput acquisition of fluorescence, brightfield and darkfield images of biological cells. Here, Blasi *et al.* demonstrate that applying machine learning algorithms on brightfield and darkfield images can detect cellular phenotypes without the need for fluorescent stains, enabling label-free assays.

## Graphical abstract

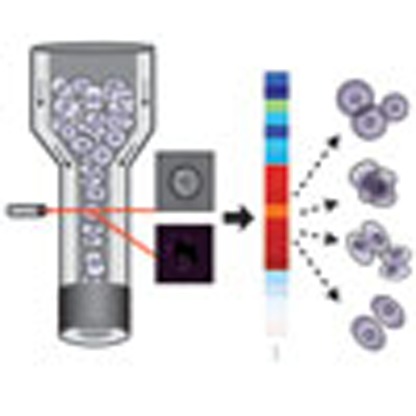

A major challenge in many modern biological laboratories is obtaining information-rich measurements of cells in high-throughput and at single-cell resolution. Conventional flow cytometry is a widespread and powerful technique for the measurement of cell phenotype and function using targeted fluorescent stains^1^. It is highly suited to the study of cell populations and rare subset identification due to its high-throughput, multi-parameter nature. The fluorescent stains can be used to label cellular components or processes, revealing specific cell phenotypes in the population and quantifying the particular state of each cell^2^. For example, quantifying the proportion of cells in each phase of the cell cycle, including mitotic phases is very useful in the modern biological laboratory^3^. It can be achieved with conventional flow cytometry using multiple stains: typically, a stoichiometric fluorescent stain for DNA reports the cells' position within the G1, S and G2 phases of the cell cycle^2^, and additional stains are needed to sort mitotic cells into phases. Often these stains are incompatible with live cell analysis (for example, antibodies against histone modifications^3^) and even if live cell reporters are available^4^ these may have confounding effects on the cells. For example, the commonly used Hoechst 33342 stain, which binds to the minor groove of the double-stranded DNA can induce single-strand DNA breaks^5^, or DRAQ5 (deep red fluorescing bisalkylaminoanthraquinone) the nuclear stain that intercalates with the cell's DNA can influence chromation organization and lead to histone dissociation^6^. Also, several different markers are usually required to unambiguously identify all cell cycle phases^7^. Therefore, an assay that reduces or even eliminates the number of stains required to identify phenotypes such as the position in the cell cycle is particularly attractive.

In recent years, the two technologies of fluorescence microscopy and flow cytometry have been integrated to create imaging flow cytometry^8^, where an image is captured of each cell as it flows past an excitation source and a CCD detector. It combines conventional flow cytometry's high-throughput speed and easy identification of each individual cell with the fluorescence microscopy's spatial image acquisition. Therefore, imaging flow cytometry measures not only fluorescence intensities but also the spatial image of the fluorescence together with brightfield and darkfield images of each cell in a population. The rich information captured using imaging flow cytometry makes it an ideal candidate for the use of high content approaches to identify complex cell phenotypes such as the cell cycle phase of an individual cell. We have previously demonstrated that measuring the shape of the nucleus from cells stained with a nuclear marker using imaging flow cytometry drastically improves the classification of mitotic phases^9^. However, the even richer morphological information that can be extracted using imaging software tools^10^ offers the prospect of using more advanced multivariate analysis techniques to mine the data and to identify various cell phenotypes, as has been successfully done for traditional microscopy images^11^,^12^,^13^,^14^. This type of analysis is also usually more accurate and less subjective than any manual analysis of the acquired images^13^ as well as more robust than typical gating strategies that rely on only few features of the cells.

Here we report that quantitative image analysis of two largely overlooked channels; brightfield and darkfield, both readily collected by imaging flow cytometers that enables cell cycle-related assays without needing any fluorescence biomarkers. We use image analysis software^9^ to extract numerical measurements of cell morphology from the brightfield and darkfield images, and then we apply supervised machine-learning algorithms to identify cellular phenotypes of interest, in the present case, cell cycle phases. The designed workflow is open-source and freely available (visit www.cellprofiler.org/imagingflowcytometry) and accompanied by step-by-step tutorials and example data sets online. Avoiding fluorescent stains provides several benefits: it reduces effort and cost, avoids potentially confounding side effects of live cell markers and frees up the remaining available fluorescence channels of the imaging flow cytometer that can be used to investigate other biological questions.

## Results

### Label-free analysis workflow

**Figure 1:**
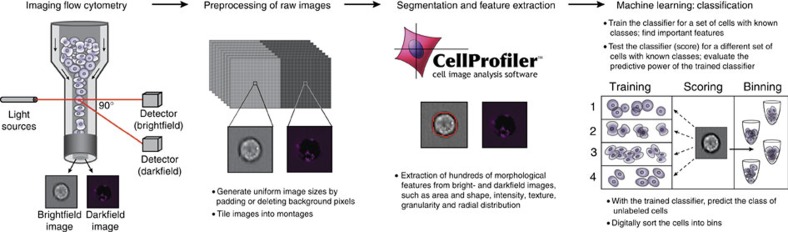
Label-free imaging flow cytometry workflow. First the brightfield and darkfield images of the cells are measured by an imaging flow cytometer. The brightfield and darkfield images depict the light transmitted through the cell and light scattered from the cells within a cone centered at a 90° angle, respectively. Then the images are preprocessed, where we reshape the images to have their sizes coincide and tile them to montages of 15 × 15 images. The montages are loaded into the open-source image software CellProfiler that we use to segment the cells' brightfield images and to extract morphological features from the images. Finally, we apply supervised machine learning such as classification. For this purpose we need an annotated set of cells where the actual cell state is known to train the classifier and to test its predictive power. Once the classifier is trained it is used to predict the state of unlabelled cells and to digitally sort the cells into bins.

The first step in the workflow of label-free cell cycle classification (Fig. 1) is to acquire brightfield and darkfield images from the cells (see Methods section). To allow visual inspection and to optimize the file size for processing, we tile individual cells' brightfield and the darkfield images into 15 × 15 montages, with up to 225 cells per montage. Then, we load the montages into the open-source imaging software CellProfiler^9^ for processing (see Methods section). There is sufficient contrast between the cells and the flow media to robustly segment the cells in the brightfield images without the need for any stains. We extract 213 features from the segmented brightfield and the full darkfield image (Supplementary Table 1). The features can be summarized into five categories: size and shape, granularity, intensity, radial distribution and texture. These image features are the input for supervised machine learning, namely classification and regression (see Methods section), which we use to predict each cell's DNA content and the mitotic phases in the cell cycle without the need for any stains. The machine-learning algorithms have to be trained on an annotated subset of the investigated cells where the true cell state, that is, the ‘ground truth' is known. The ground truth can be obtained either by manual identification (by a trained biologist or using software tools^11^) or from labelling a subset of the investigated cells with fluorescent stains (see Methods section).

### Cell cycle analysis of fixed Jurkat cells

As an initial demonstration of our technique, we sought a label-free way to measure important cell cycle phenotypes including a continuous property (a cell's DNA content, from which G1, S and G2 phases can be estimated) and discrete phenotypes (the mitotic phase of a cell: prophase, anaphase, metaphase and telophase). We used the ImageStream platform to capture images of 32,255 asynchronously growing Jurkat cells (Supplementary Fig. 1). As controls, the cells were fixed and stained with PI (propidium iodide) to quantify DNA content and a MPM2 (mitotic protein monoclonal #2) antibody to identify mitotic cells (Supplementary Fig. 2). These fluorescent markers were used to annotate a subset of the cells with the ground truth (expected results) needed to train the machine-learning algorithms and to evaluate the predictive accuracy of our label-free approach (see Methods section). Since it is infeasible to accurately identify individual cells in the G1, S and G2 phase based only on one nuclear marker^5^, we do not aim to predict those phases individually but to predict each cell's DNA content. Subsequently, we use the Watson pragmatic curve fitting algorithm^15^ (see Methods section) to estimate the percentage of cells in each of the G1/S/G2M phases based on the predicted DNA content.

**Figure 2:**
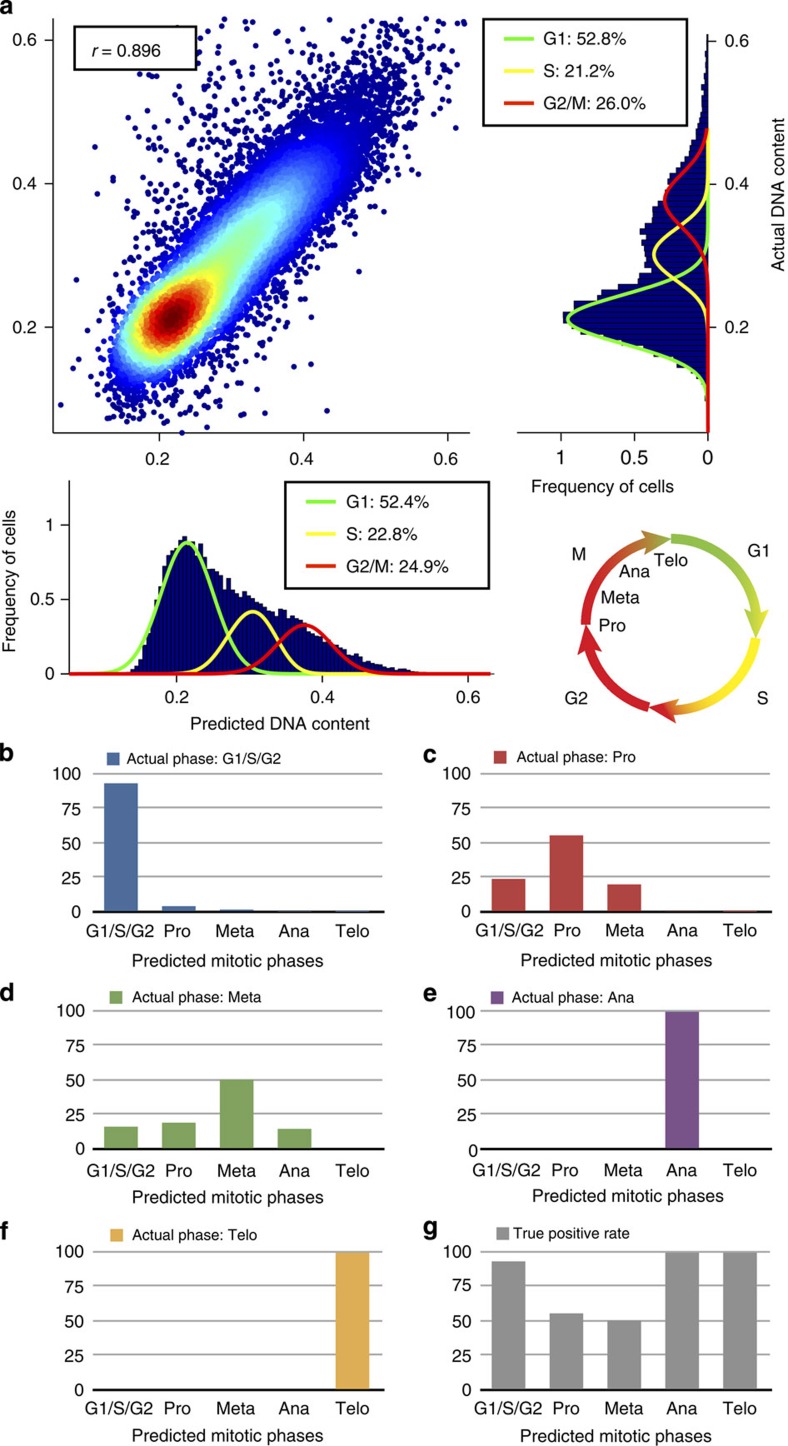
Machine learning allows for robust label-free prediction of DNA content and cell cycle phases of Jurkat cells. (**a**) We find a Pearson's correlation of *r=*0.896±0.007 (error bars indicate the s.d. obtained via 10-fold cross-validation) between actual DNA content and predicted DNA content based on regression using brightfield and darkfield morphological features only (see Methods section). We used the Watson pragmatic curve fitting algorithm to specify the fraction of cells in the G1, S and G2 phases. (**b**–**f**) For cells that are actually in a particular phase (for example, **b** shows cells in G1/S/G2), the bar plots show the classification results based on brightfield and darkfield morphological features only (for example, **b** shows that the few cells in prophase (Pro), metaphase (Meta), anaphase (Ana), and telophase (Telo) are errors). (**g**) Bar plot of the true positive rates of the cell cycle classification.

Using only cell features measured from brightfield and darkfield images, we were able to devise a regression ensemble (using least squares boosting^16^) that accurately predicts each cell's DNA content, obtaining a Pearson's correlation of *r*=0.896±0.007 (error bars indicate the s.d. obtained via 10-fold (*n*=10) cross-validation here and in all following statements of the Results section unless stated differently; see Methods section and Supplementary Note 1) between predicted and actual nuclear stain intensity (Fig. 2a). This highly accurate prediction of the DNA content can be used to further categorize G1, S and G2/M cells or to allocate each cell a time position within the cell cycle via the ergodic rate analysis, where cells are sorted according to their DNA content^17^. Moreover, we were able to classify mitotic phases (using random undersampling^18^ to compensate for the high class imbalance) with true positive rates of 55.4±7.0% (for prophase), 50.2±17.2% (for metaphase), 100% (for anaphase and telophase) and 93.1±0.5% for the non-mitotic phases (Fig. 2b–g and Supplementary Table 2). We analysed which features have the most significant contributions for the prediction of both the nuclear stain and the mitotic phases by ‘leave one out' cross-validation (Supplementary Table 3). We find that leaving one feature out has only a minor effect on the results of the supervised machine-learning algorithms we used, likely because many features are highly correlated to others. The most important features are intensity, area, shape and radial distribution of the brightfield images.

### Detection of mitotic phase block

**Figure 3:**
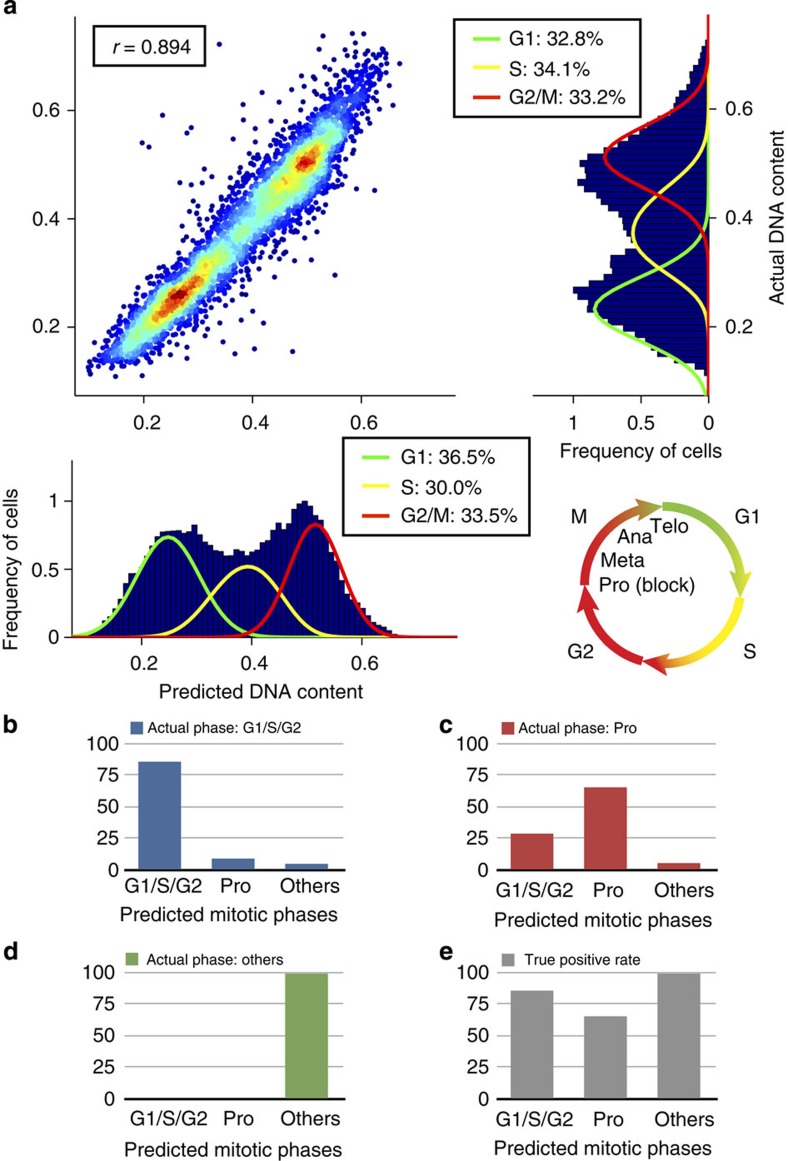
Label-free prediction of DNA content and cell cycle phases for fixed Jurkat cells treated with a prophase blocking agent. (**a**) Based only on brightfield and darkfield features, we find a Pearson's correlation of *r*=0.894±0.032 (error bars indicate the s.d. obtained via 10-fold cross-validation) between actual DNA content and predicted DNA content using regression (see Methods section). We applied the Watson pragmatic algorithm to determine the G1, S and G2/M phases in the DNA histograms. (**b**–**d**) For cells that are actually in a particular phase (for example, **b** shows cells in G1/S/G2), the bar plots show the classification results (see Methods section) (for example, **b** shows that the few cells in prophase (Pro) and the other mitotic phases (others) are errors). Note that we grouped the cells in metaphase, anaphase and telophase into one class since we only detected very little cells in those phases after treatment with the prophase blocking agent. (**e**) Bar plot of the true positive rates of the cell cycle classification. Using boosting with random undersampling to compensate for class imbalances, we obtain true positive rates of 65.5±6.3% (P), 85.8±1.4% (G1/S/G2) and 100% (others).

The assessment of the therapeutic blocking of the cell cycle (in a particular phase) is of particular importance. We tested the method's ability to predict the DNA content of Jurkat cells treated with 50 μM Nocodazole, a mitotic blocking agent. To confirm the magnitude of the block of cells in mitosis, we performed three additional replicates demonstrating an average increase of cells in the G2/M phase of 19.0±11.0% (error bars indicate the s.d. obtained from *n*=3 replicates for each condition) compared with the control (Supplementary Fig. 3). The label-free prediction of the DNA content has a Pearson's correlation of *r*=0.894±0.032 with the true DNA content (PI is used as a fixed cell nuclear stain to provide the ground truth for the machine-learning algorithms) and the percentage of cells in the G1, S and G2/M phases are in excellent agreement (Fig. 3a). Therefore, the technique is successfully detecting the expected increase in the G2/M cells due to the blocking agent based on the predicted DNA content. Again, we were able to classify mitotic phases and found true positive rates of 65.5±6.3% (for prophase), 100% (for the other mitotic phases) and 85.8±1.4% for the non-mitotic phases (Fig. 3b–e and Supplementary Table 4). Treatment of the cells with the mitotic blocking agent led to an increase in the percentage of prophase cells from 1.88 to 11.07, which is confirmed by comparison with the ground truth (Supplementary Table 2 and Table 4) and in agreement with the identified magnitude of the block of cells in mitosis.

### Cell cycle analysis of live Jurkat cells

**Figure 4:**
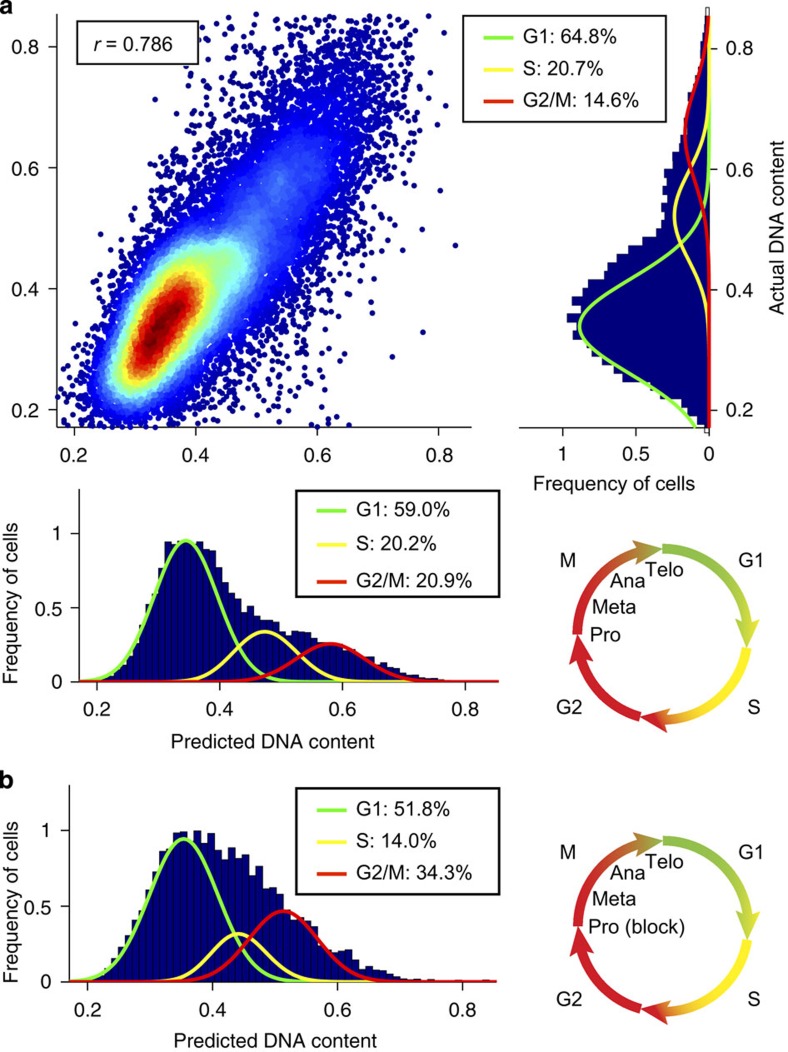
Label-free prediction of DNA content for live Jurkat cells and detection of a phase blockage. (**a**) Supervised machine learning (trained using live cells stained with DRAQ5 to determine the DNA content) allows for robust label-free prediction of the DNA content of live cells based only on brightfield and darkfield images. We find a Pearson correlation of *r*=0.786±0.010 (error bars indicate the s.d. obtained via 10-fold cross-validation) between actual DNA content and predicted DNA content using regression (see Methods section). We believe this reduction in correlation from the value of 0.896 obtained for fixed cells to be a consequence of the greater variability of the uptake of the live DNA dye compared with the staining achieved with fixed cells. Despite the reduction in correlation a value of 0.786 is still high enough to make this a viable method for the cell cycle analysis of live cells. As previously, we determine the fraction of cells in the G1, S and G2/M phases using the Watson pragmatic curve fitting algorithm. (**b**) We predict an increase of 13.4% in the G2/M phase after the cells were treated with 50μM Nocodazole, which is in good agreement with the average increase of 19.0±11.0% in G2/M as was found for three independent cell populations under the same treatment (Supplementary Figure 3). The phase-blocked data set was not labelled with any marker. Instead, we trained our machine learning algorithm on the untreated data set, which was labelled with a DRAQ5 DNA stain (see **a**) and used the trained machine learning algorithm to predict the DNA stain of the blocked cells.

Many experimental protocols require live cells rather than fixed. We tested the ability of the technique to detect cell cycle changes in live Jurkat cells. To provide ground truth (that is, the expected cell cycle distribution), the cells were stained with DRAQ5, a live-cell DNA stain (Fig. 4a). Like most live-cell-compatible DNA stains, DRAQ5 is not an ideal marker because of the variability of uptake of the dye in live cells^19^, nonetheless, we obtain a Pearson's correlation of *r*=0.786±0.010 for predicting the DNA content of untreated cells. With a regression ensemble trained on the stained live cells, we are also able to predict the effect of treatment with a phase-blocking agent on an entirely unstained data set (Fig. 4b). We detect an increase of cells in the G2/M phase from 20.9 to 34.3% when the cells are treated with 50 μM Nocodazole; this is consistent with the average increase of 19.0±11.0% obtained from repeating the phase block experiments with stained cells (Supplementary Fig. 3).

### Cell cycle analysis of fission yeast

**Figure 5:**
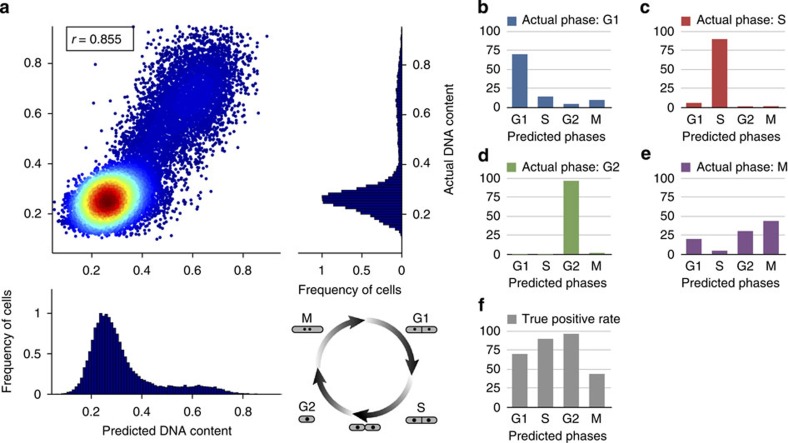
Label-free prediction of DNA content and cell cycle phases for fission yeast cells. (**a**) Based only on brightfield and darkfield features, we find a Pearson's correlation of *r*=0.855±0.006 (error bars indicate the s.d. obtained via 10-fold cross-validation) between actual DNA content and predicted DNA content using regression (see Methods section). Note that the fission yeast cell cycle is different from the Jurkat cell cycle since the two daughter cells divide between the S and G2 phases (and not at the end of M phase as is the case for Jurkat cells). (**b**–**e**) For cells that are actually in a particular phase (for example, **b** shows cells in G1), the bar plots show the classification results (see Methods section) (for example, **b** shows that the cells in S, G2 and M are errors). (**f**) Bar plot of the true positive rates of the cell cycle classification. Using boosting with random undersampling to compensate for class imbalances, we obtain true positive rates of 70.2±2.2% (G1), 90.1±1.1% (S), 96.8±0.3% (G2) and 44.0±8.4 (M).

To explore the generality of our method for other cell types, we tested it on another species, fission yeast (Supplementary Fig. 4). The yeast cells were fixed and stained with PI to measure the DNA content of each cell (see Methods section); subsequently the cells were assigned to the G1, S, G2 or M phase by manually gating on image based metrics from the PI channel of the Imagestream data^20^, which provided the ground truth (Supplementary Fig. 5). The label-free regression predicts a DNA content with a Pearson's correlation of *r*=0.855±0.006 (Fig. 5a) and a classification accuracy of 70.2±2.2% (G1), 90.1±1.1% (S), 96.8±0.3% (G2) and 44.0±8.4% (M) (Fig. 5b–f and Supplementary Table 5).

## Discussion

We demonstrate here that it is possible to determine a cell population's DNA content and mitotic phases based entirely on features extracted from cells' brightfield and darkfield images, as obtained in high-throughput via imaging flow cytometry. The method requires an annotated data set to train the machine-learning algorithms, either by staining a subset of the investigated cells with markers, or by visual inspection and assignment of cell classes of interest. Once the machine-learning algorithm is trained for a particular cell type and phenotype, the consistency of imaging flow cytometry allows high-throughput scoring of unlabelled cells for discrete and well-defined phenotypes (for example, mitotic cell cycle phases) and continuous properties (for example, DNA content).

The same basic strategy can be readily adapted to measure other phenotypes, making this a generally useful approach for label-free, single-cell phenotyping in the modern biological laboratory. The method can also be used retrospectively on data sets that do not have the necessary stains for phenotype identification, providing an annotated data set is available to train the algorithms (see Methods section). While current imaging flow cytometers do not have physical cell-sorting capabilities, and for now our approach is suited to experimental contexts where samples are analysed only, this approach may offer the possibility to entirely avoid any fluorescent stain and opens up the perspective for a new generation of image flow cytometers that could operate without fluorescence channels.

The workflow we designed is open-source and freely available (www.cellprofiler.org/imagingflowcytometry and Supplementary Note 1). Label-free identification of phenotypes enables continuous, non-destructive monitoring of cell samples, minimizes potentially confounding influences of the stains on the cells and maximizes available fluorescence channels to investigate biological questions such as the search for novel hallmarks in cell cycle^21^, the identification of stem and progenitor cells^22^ or the proliferation of cancer cells^23^.

## Methods

### Code availability

All processing steps are described in a step-by-step tutorial hosted on an up-to-date website with guidance on carrying out the tutorial (visit www.cellprofiler.org/imagingflowcytometry; a static version of the tutorial can also be found in Supplementary Note 1). The code and the analysed data are freely available on the webpage. The code is also available as Supplementary Information (Supplementary Code 1–6). We used Matlab version 8.0.0.783 (R2012b)) and CellProfiler version 2.1.1 for our analysis.

### Cell culture and phase block

Ten million E6.1 Jurkat Cells (Fred Hutchinson Cancer Research Center derived clone, Cell Services, CRUK) were cultured in RPMI media (Cat no 31870-082, Life Technologies, Inc., USA) containing 10% FBS, Penicillin/Streptomycin/Glutamine (Sigma-Aldrich G6784) at 1% and 2-Mercaptoethanol (50 μM) at 37 °C/5% CO2. For cells requiring a phase block the cells were incubated with 50 μM Nocodazole for 20 h at 37 °C per 5% CO_2_, counted and checked for viability using a Vi-Cell counter (Beckman Coulter, Inc., USA). Cells were washed once in PBS containing 2% FBS (wash buffer) and the cellular suspensions were divided in two.

### Live cells

Half of the cells were resuspended in 100 μl of wash buffer and DRAQ5 (Cat no DR50200, Biostatus) added to a final concentration of 5 μM before running on the ImageStream X.

### Fixed cells

The other half of cells was fixed in 70% ethanol for at least 1 h. After fixation, the cells were washed once in wash buffer and treated with 0.1% Triton X-100 (Cat no X100-100 ML, Sigma, USA) for 10 min. Cells were spun down and incubated for 1 h with anti-phospho-Ser/Thr-Pro, MPM2 Cy5 conjugate MPM2 (1:100, Cat no 16-220, Millipore, USA) made up in PBS containing 0.2% Tween (Cat no 27,434-8, Sigma, USA) and 0.1% BSA (Cat no A4503-100G, Sigma, USA). Cells were washed once in wash buffer and stained with a 10 μg ml^−1^ PI (Cat no P4170, Sigma, USA) and 11 μg ml^−1^ Ribonuclease A (Cat no R5125, Sigma, USA) solution made up in PBS (100 μl). Cells were stained with PI for at least 30 min and run on the Imagestream X.

### Fission yeast

Cell culture conditions and growth media were as previously described by Moreno *et al.*^24^ PN1 (wild-type haploid, strain 972h− mating type, lab stock) cells were grown in YE4S media and maintained in exponential phase. ∼5 × 10^6^ cells were harvested for fixation in 70% ice cold ethanol before storage at 4 °C. Cells were then washed and resuspended in 1 ml of 50 mM sodium citrate, treated with 0.1 mg ml^−1^ RNase A (Sigma-Aldrich, UK) at 37 °C overnight. Subsequently cells were stained with PI (2 μg ml^−1^) and FITC (2 μg ml^−1^) before sonication (∼20 s) using a sonication probe (JSP, Inc., USA). Cells were then resuspended in a volume of 500 μl, before running on the Imagestream X. Subsequent cell cycle stage assignment was performed as described by Patterson *et al.*^20^ In brief, this assignment is based on a combination of morphometric and intensity features extracted from PI images. Low PI intensity cells containing two nuclei are defined as G1. High PI intensity cells containing two nuclei containing cells are defined as S. Elongated, low intensity PI, uni-nucleate cells are defined as G2. Elongated, high intensity PI, single cells are defined as M phase (Supplementary Fig. 5).

### Curve fitting for DNA histograms

We used the Watson pragmatic algorithm (Supplementary Code 6) to obtain probability distributions for the cells being in G1, S and G2/M phase of cell cycle.

### Image acquisition by imaging flow cytometry

We used the ImageStream X platform to capture images of both live and fixed asynchronously growing Jurkat cells. For each cell, we captured images of brightfield and darkfield as well as fluorescent channels to measure the PI that quantifies DNA content and a MPM2 antibody to identify cells in mitosis. After image acquisition, we used the IDEAS analysis tool (this is software that accompanies the ImageStream X software) to discard multiple cells or debris, omitting them from further analysis, as described in the tutorial (Supplementary Note 1). The resulting data are provided on www.cellprofiler.org/imagingflowcytometry.

### Typical ImageStream settings

Sample volume: 2.6 μl (extracted from the 100 μl loaded). Flow diameter: 7 μm. Velocity of flow: 44 m s^−1^. Resolution: 0.5 μm. Magnification: × 60. Camera sensitivity: 256 on all channels. Camera gain: 1. Brightfield LED intensity: 88 mW. Darkfield laser intensity: 1 mW. 488 nm laser intensity: 25 mW. 642 nm laser intensity: 150 mW.

### Image processing

The image sizes from the ImageStream cytometer range between 30 × 30 and 60 × 60 pixels (data provided on www.cellprofiler.org/imagingflowcytometry). We reshape their sizes to 55 × 55 pixel images by either adding pixels with random values that we sampled from the background of the image for images that are smaller or by discarding pixels on the edge of the image for images that are too large. We note that the discarded pixels are only from the image background and not from the segmented cell. This procedure therefore does not affect the analysis. To demonstrate this we analysed if discarding pixels from larger images has an effect on the results on our method (Supplementary Table 6) and found robust results over a broad parameter range. Only if we reshape the images to sizes that are smaller than the cells' diameter (that is, parts of the cells get cropped) does the quality of the method decrease. We then tile the images to 15 × 15 montages, with up to 225 cells per montage. Example montages are provided (data provided on www.cellprofiler.org/imagingflowcytometry). A script to create the montages is provided (Supplementary Code 1) and its use is described in the tutorial (Supplementary Note 1) and on the webpage.

### Segmentation and feature extraction

We load the image montages of 15 × 15 cells into the open-source image software CellProfiler (version 2.1.1). The darkfield image shows light scattered from the cells within a cone centred at a 90° angle and hence does not necessarily depict the cell's physical shape nor does it align with the brightfield image. Therefore, we do not segment the darkfield image but instead use the full image for further analysis. In the brightfield image, there is sufficient contrast between the cells and the flow media to robustly segment the cells. We segment the cells in the brightfield image by enhancing the edges of the cells and thresholding on the pixel values. We then extract features, which we categorized into size and shape, granularity, intensity, radial distribution and texture. The CellProfiler pipeline to carry out all of these steps is provided (Supplementary Code 2). The measurements are exported in a text file, an example of which is provided (data provided on www.cellprofiler.org/imagingflowcytometry). The measurements are post-processed using a script to discard cells with missing values (Supplementary Code 3). The use of these steps is described in the tutorial (Supplementary Note 1) and on the webpage.

### Determination of ground truth

To train the machine learning algorithm we need a subset of cells where the cell's true state is annotated, that is, the ground truth is known. For the experiment shown in Fig. 1, the cells were labelled with a PI and a MPM2 stain. As the ground truth (expected results) for the cells' DNA content, we extracted the integrated intensities of the nuclear PI stain with the imaging software CellProfiler (Supplementary Code 2). The mitotic cell cycle phases were identified with the IDEAS analysis tool by categorizing the MPM2-positive cells into anaphase, prophase and metaphase using a limited set of user-formulated morphometric parameters (Supplementary Figure 2) on their PI stain images followed by manual confirmation. The telophase cells were identified using a complex set of masks (using the IDEAS analysis tool) on the brightfield images to gate doublet cells. We used those values as the ground truth to train the machine learning algorithm and to evaluate the prediction of the nuclear stain intensity.

We note that the ground truth is measured using the same modality, that is, the ImageStream system; this preserves the consistency of the presentation of the cell for measurement. If we seeded the cells on a plate to use microscopy the cell's shape and morphology would be very different. However, the method we describe here could equally well be used to determine cell phenotypes from brightfield and darkfield images from traditional microscopy provided the ground truth is measured using the same microscopy platform. For the analysis of short-lived mitotic phases then large numbers of cells would be required; however, this should not be problematic given the development of high-throughput imaging systems. The advent of three-dimensional high-resolution microscopy has given rise to images with an even richer information content and provided enough cells could be measured then these systems would make good candidates for the method proposed here.

### Machine learning

For the prediction of the DNA content, we use LSboosting as implemented in Matlab's fitensemble routine (Supplementary Code 4). For the assignment of the mitotic cell cycle phases, we use RUSboosting as also implemented in Matlab's fitensemble routine (Supplementary Code 5). In both cases, we partition the cells into a training and a testing set. The brightfield and darkfield features of the training set as well as the ground truth of these cells are used to train the ensemble. Once the ensemble is trained, we evaluate its predictive power on the testing set. To demonstrate the generalizability of this approach and to obtain error bars for our results the procedure is 10-fold cross-validated. To prevent overfitting the data the stopping criterion of the training was determined via fivefold internal cross-validation. All of these steps are described in the tutorial (Supplementary Note 1) and on the webpage.

In addition, we analysed which features have the most significant contributions for the prediction of both the nuclear stain and the mitotic phases by ‘leave one out' cross-validation (Supplementary Table 3). We find that leaving one feature out has only a minor effect on the results of the supervised machine learning algorithms we used, likely because many features are highly correlated to others. The most important features are intensity, area and shape and radial distribution of the brightfield images.

### Retrospective data analysis

The described method can be used retrospectively to analyze data that was not originally acquired with label-free phenotype identification in mind. As demonstrated in the paper, either of two requirements must be met: (1) the phenotype of interest must be recognizable by eye or quantifiable/classifiable by image analysis, given the existing label-free images, or (2) the phenotype must be recognizable by eye or quantifiable/classifiable by image analysis in a separately stained subset of images prepared at the same time. Either of these approaches will provide the ground truth required to train the algorithms for label-free identification from retrospective image data on cells that are otherwise identically prepared and imaged, but lacking any stains. This approach offers the possibility to study the properties of different cell phenotypes using data that previously did not allow distinguishing the phenotypes.

The same overall approach could also in theory be used to carry out label-free assays (whether retrospectively or not) using the image data from conventional microscopy as opposed to imaging flow cytometry. Adherent cells that are reasonably flattened improve the visibility of morphological features by which to determine phenotypes; however, the non-uniform presentation of each cell (versus imaging flow cytometry) is a disadvantage. Whether any particular application is feasible would be an empirical question.

## Additional information

**How to cite this article**: Blasi, T. *et al.* Label-free cell cycle analysis for high-throughput imaging flow cytometry. *Nat. Commun.* 7:10256 doi: 10.1038/ncomms10256 (2016).

## Supplementary Material

Supplementary Figures, Supplementary Tables and Supplementary Notes Supplementary Figures 1-5, Supplementary Tables 1-6 and Supplementary Notes 1-2

Supplementary Code 1 Makes montages of 25x25 cells from the single tiff images

Supplementary Code 2 CellProfiler Pipeline that extracts features from the montages

Supplementary Code 3 Preprocesses the extracted features and converts them into a Matlab format

Supplementary Code 4 Performs the least-squares boosting regression on the data

Supplementary Code 5 Performs the reduced undersampling boosting classification on the data

Supplementary Code 6 Function to perform the Watson Pragmatic curve fitting algorithm on the intensity histograms
