# Supplementary material for: Label-free cell cycle analysis for high-throughput imaging flow cytometry
Source: Nat Commun. 2016 Jan 7;7:10256. doi: 10.1038/ncomms10256 (PMC4729834; doi:10.1038/ncomms10256)
Supplement: Supplementary Figures, Supplementary Tables and Supplementary Notes — Supplementary Figures 1-5, Supplementary Tables 1-6 and Supplementary Notes 1-2 [file ncomms10256-s1.pdf]

Supplementary Figures

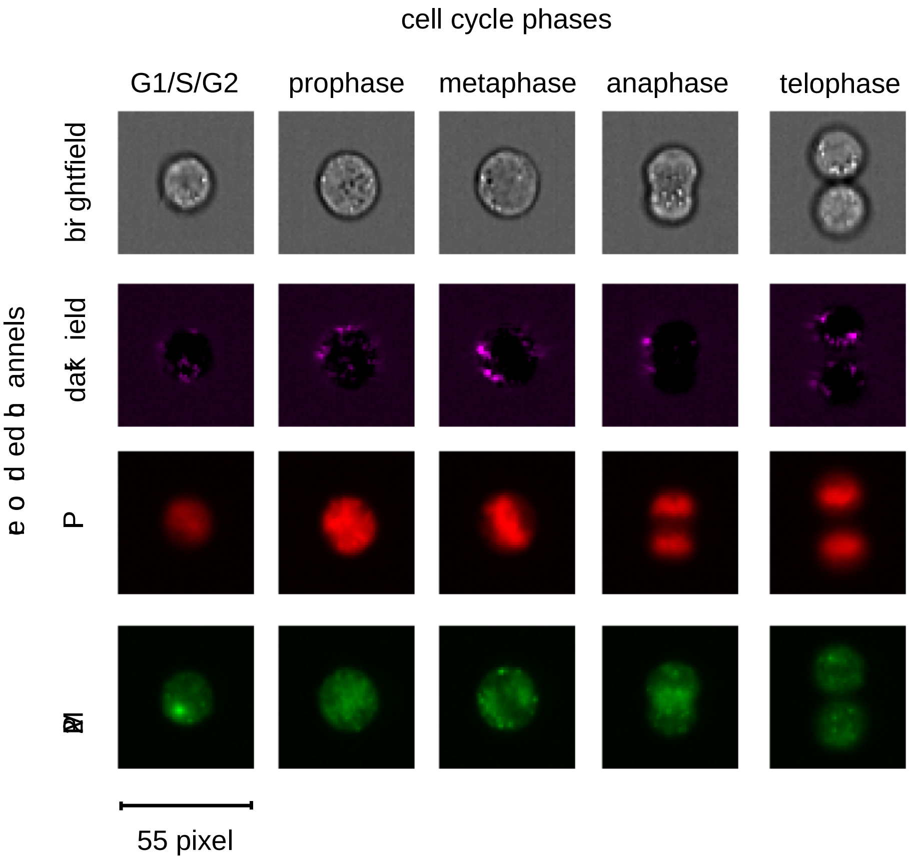

Supplementary Figure 1 | Images of Jurkat cells captured by imaging flow cytometry. Typical brightfield, darkfield, PI (propidium iodide) and MPM2 (mitotic protein monoclonal #2) antibody images of cells in the G1/S/G2 phases, prophase, metaphase, anaphase and telophase of the cell cycle. The size of the images is 55x55 pixels.

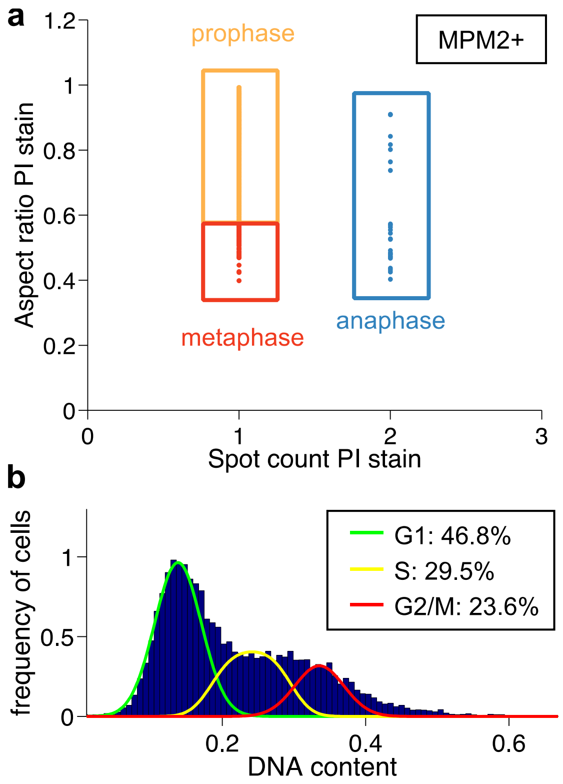

Supplementary Figure 2 | Ground truth annotation for fixed Jurkat cells. (**a**) First, MPM2 (mitotic protein monoclonal #2) negative cells were identified and removed from analysis. Then, morphological metrics on the remaining cells' PI (propidium iodide) images were used to identify prophase, metaphase and anaphase. The telophase cells were identified by gating on the aspect ratio versus area of the brightfield image to determine when the cell has formed two distinct daughter cells. These results were verified by visual inspection by the user. The resulting assignments were used to train the machine learning algorithms. (**b**) The PI intensity histogram generated using the IDEAS software. The Watson pragmatic curve fitting algorithm was used to specify the fraction of cells in the G1, S and G2 phases of the cell cycle.

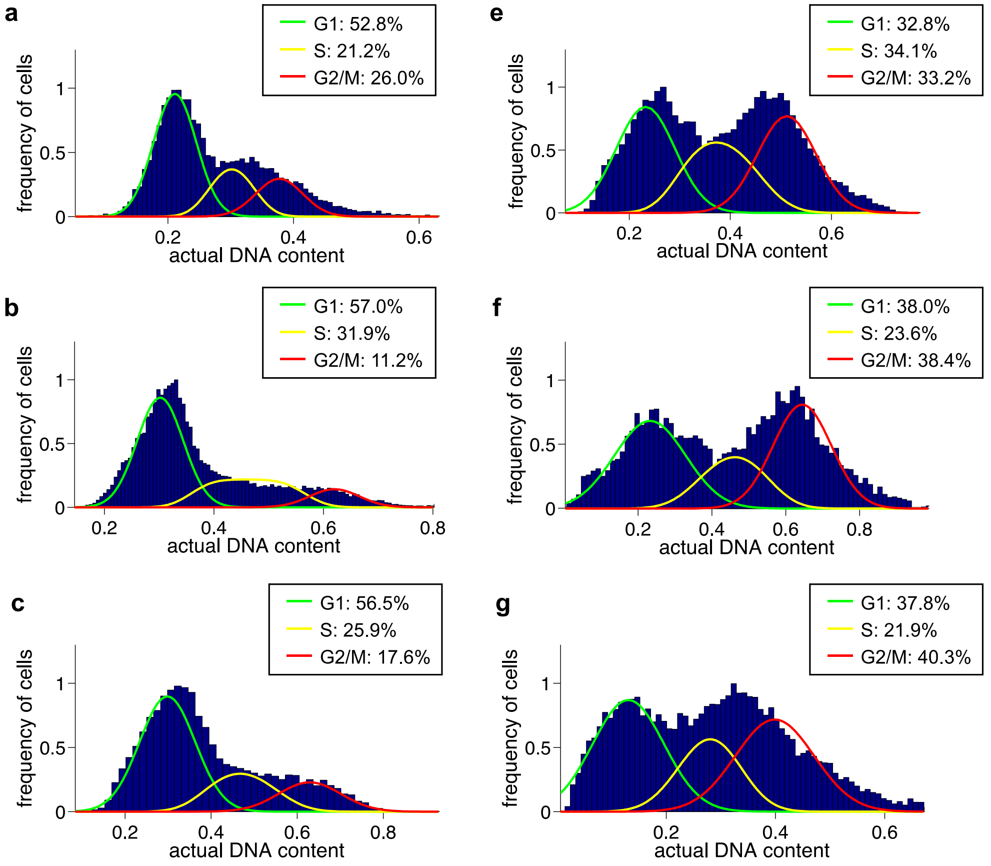

d

	mean	sd
G1:	55.4 %	2.3 %
S:	26.3 %	5.4 %
G2/M:	18.3 %	7.4 %

h

	mean	sd
G1:	36.2 %	3.0 %
S:	26.5 %	6.6 %
G2/M:	37.3 %	3.6 %

Supplementary Figure 3 | Fraction of cells in the G1, S and G2/M phases of cell cycle for three independent cell populations without treatment and after treatment with a blocking agent. (**a-c**) Assessment of cells in the G1, S and G2 cell cycle phases for three independent cell populations without treatment using the Watson pragmatic algorithm. (**d**) On average 18.3% of the cells are in G2/M while we observe a standard deviation (s.d.) of 7.4% among the n = 3 replicates. (**e-g**) Assessment of cells in the G1, S and G2 cell cycle phases for n = *3* independent cell populations after treatment with 50µM Nocadozole using the Watson pragmatic algorithm. (**h**) On average 37.3% of the cells are in G2/M (standard deviation 3.6%). The effect of the phase blockage (on average 19.0% increase in G2/M) is clearly higher than the biological variance (11.0%; i.e. the sum of the standard deviations) among the experiments.

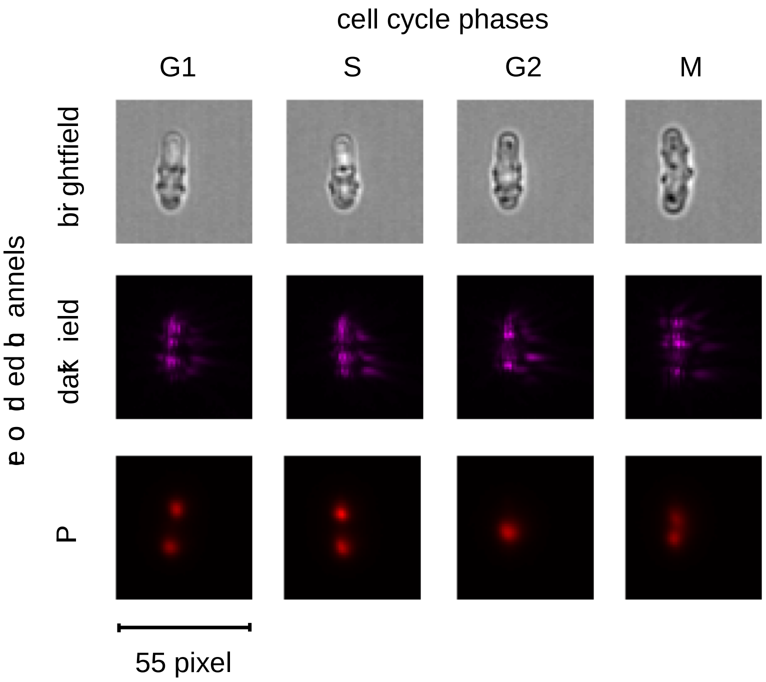

Supplementary Figure 4 | Images of fission yeast cells captured by imaging flow cytometry. Typical brightfield, darkfield and PI (propidium iodide) images of cells in the G1, S, G2 and M phases of the cell cycle. The size of the images is 55x55 pixels.

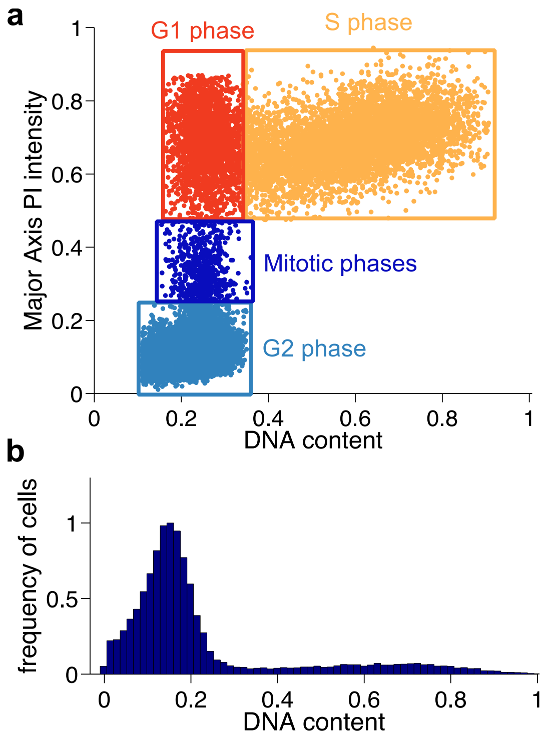

Supplementary Figure 5 | Ground truth annotation for yeast cells. Annotation of cells in G1, S, G2 and M phase on PI (propidium iodide) staining, in order to create the "ground truth" (expected results). (**a**) Morphological metrics on the cells' PI images were used to identify the G1, S, G2 and M phases. The resulting assignments were used to train the machine learning algorithms. (**b**) The PI intensity histogram generated using the IDEAS software.

Supplementary Tables

Feature class	Feature number	Feature name	Brightfield	Darkfield
Area and shape	1	AreaShape_Area	x	o
	2	AreaShape_Compactness	x	o
	3	AreaShape_Eccentricity	x	o
	4	AreaShape_Extent	x	o
	5	AreaShape_FormFactor	x	o
	6	AreaShape_MajorAxisLength	x	o
	7	AreaShape_MaxFeretDiameter	x	o
	8	AreaShape_MaximumRadius	x	o
	9	AreaShape_MeanRadius	x	o
	10	AreaShape_MedianRadius	x	o
	11	AreaShape_MinFeretDiameter	x	o
	12	AreaShape_MinorAxisLength	x	o
	13	AreaShape_Perimeter	x	o
Zernike polynomials	14	AreaShape_Zernike_0_0	x	o
	…	…	x	o
	43	AreaShape_Zernike_9_9	x	o
Granularity	44	Granularity_1	x	x
	…	…	x	x
	48	Granularity_5	x	x
Intensity	49	Intensity_IntegratedIntensityEdge	x	x
	50	Intensity_IntegratedIntensity	x	x
	51	Intensity_LowerQuartileIntensity	x	x
	52	Intensity_MADIntensity	x	x
	53	Intensity_MassDisplacement	x	x
	54	Intensity_MaxIntensityEdge	x	x
	55	Intensity_MaxIntensity	x	x
	56	Intensity_MeanIntensityEdge	x	x
	57	Intensity_MeanIntensity	x	x
	58	Intensity_MedianIntensity	x	x
	59	Intensity_MinIntensityEdge	x	x
	60	Intensity_MinIntensity	x	x
	61	Intensity_StdIntensityEdge	x	x
	62	Intensity_StdIntensity	x	x
	63	Intensity_UpperQuartileIntensity	x	x
Radial distribution	64	RadialDistribution_FracAtD_1	x	x
	65	RadialDistribution_FracAtD_2	x	x
	66	RadialDistribution_FracAtD_3	x	x
	67	RadialDistribution_FracAtD_4	x	x
	68	RadialDistribution_MeanFrac_1	x	x
	69	RadialDistribution_MeanFrac_2	x	x
	70	RadialDistribution_MeanFrac_3	x	x
	71	RadialDistribution_MeanFrac_4	x	x
	72	RadialDistribution_RadialCV_1	x	x
	73	RadialDistribution_RadialCV_2	x	x
	74	RadialDistribution_RadialCV_3	x	x
	75	RadialDistribution_RadialCV_4	x	x
	76	Texture_AngularSecondMoment_3_0	x	x
Texture	77	Texture_AngularSecondMoment_3_135	x	x
	78	Texture_AngularSecondMoment_3_45	x	x
	79	Texture_AngularSecondMoment_3_90	x	x
	80	Texture_Contras_3_0	x	x
	81	Texture_Contras_3_135	x	x
	82	Texture_Contras_3_45	x	x
	83	Texture_Contras_3_90	x	x
	84	Texture_Correlation_3_0	x	x
	85	Texture_Correlation_3_135	x	x
	86	Texture_Correlation_3_45	x	x
	87	Texture_Correlation_3_90	x	x
	88	Texture_DifferenceEntropy_3_0	x	x

	89	Texture_DifferenceEntropy_3_135	x	x
	90	Texture_DifferenceEntropy_3_45	x	x
	91	Texture_DifferenceEntropy_3_90	x	x
	92	Texture_DifferenceVariance_3_0	x	x
	93	Texture_DifferenceVariance_3_135	x	x
	94	Texture_DifferenceVariance_3_45	x	x
	95	Texture_DifferenceVariance_3_90	x	x
	96	Texture_Entropy_3_0	x	x
	97	Texture_Entropy_3_135	x	x
	98	Texture_Entropy_3_45	x	x
	99	Texture_Entropy_3_90	x	x
	100	Texture_Gabor	x	x
	101	Texture_InfoMeas1_3_0	x	x
	102	Texture_InfoMeas1_3_135	x	x
	103	Texture_InfoMeas1_3_45	x	x
	104	Texture_InfoMeas1_3_90	x	x
	105	Texture_InfoMeas2_3_0	x	x
	106	Texture_InfoMeas2_3_135	x	x
	107	Texture_InfoMeas2_3_45	x	x
	108	Texture_InfoMeas2_3_90	x	x
	109	Texture_InverseDifferenceMoment_3_0	x	x
	110	Texture_InverseDifferenceMoment_3_135	x	x
	111	Texture_InverseDifferenceMoment_3_45	x	x
	112	Texture_InverseDifferenceMoment_3_90	x	x
	113	Texture_SumAverage_3_0	x	x
	114	Texture_SumAverage_3_135	x	x
	115	Texture_SumAverage_3_45	x	x
	116	Texture_SumAverage_3_90	x	x
	117	Texture_SumEntropy_3_0	x	x
	118	Texture_SumEntropy_3_135	x	x
	119	Texture_SumEntropy_3_45	x	x
	120	Texture_SumEntropy_3_90	x	x
	121	Texture_SumVariance_3_0	x	x
	122	Texture_SumVariance_3_135	x	x
	123	Texture_SumVariance_3_45	x	x
	124	Texture_SumVariance_3_90	x	x
	125	Texture_Variance_3_0	x	x
	126	Texture_Variance_3_135	x	x
	127	Texture_Variance_3_45	x	x
	128	Texture_Variance_3_90	x	x

Supplementary Table 1 | List of morphological features extracted from brightfield and darkfield images. We used the imaging software CellProfiler to extract six different classes of features: Area and shape, Zernike polynomials, granularity, intensity, radial distribution and texture. Features that were taken for either the brightfield or the darkfield are marked with x, whereas features that were not measured are marked with o (e.g., features that require segmentation were not measured for the darkfield images). For details on the calculation of the features we refer to the online manual of the CellProfiler software (www.cellprofiler.org).

Predicted cell cycle phases

Actual cell cycle phases

	G1/S/G2	Prophase	Metaphase	Anaphase	Telophase	Fraction of population
G1/S/G2	93.13	4.20	1.75	0.37	0.55	97.79
Prophase	23.64	56.03	19.67	0.17	0.50	1.88
Metaphase	17.65	19.12	50.00	13.42	0	0.21
Anaphase	0	0	0	100	0	0.05
Teleophase	0	0	0	0	100	0.08

Actual

Supplementary Table 2 | Detailed results of cell cycle phase prediction. This display indicates the types of errors made by the system (i.e. the confusion matrix of the classification). For example, 97.8% of all cells in the sample were in G1/S/G2. 93.13% of these were correctly classified (predicted) by the machine learning system as in G1/S/G2. Of the ~7.8% incorrectly classified, most were miss-classified as prophase – not surprisingly, given that humans are inconsistent about where exactly G2 ends and prophase begins. By contrast, all cells that were actually in anaphase and telophase were correctly classified (predicted) in the proper phase by the machine learning system. Prophase and metaphase are less accurate, and are often confused with each other and with G1/S/G2.

	Left out features	Correlation in prediction of DNA content	Average true positive rate for prediction of mitotic phases
Brightfield	Area and shape	0.895	90.2%
	Zernike polynomials	0.898	92.1%
	Granularity	0.896	92.3%
	Intensity	0.888	90.8%
	Radial distribution	0.886	91.5%
	Texture	0.894	92.6%
Darkfield	Granularity	0.896	92.2%
	Intensity	0.896	92.0%
	Radial distribution	0.896	92.9%
	Texture	0.896	92.7%
	(none)	0.896	92.3%
	All brightfield features	0.758	81.8%
	All darkfield features	0.889	91.3%

Supplementary Table 3 | Importance of morphological features in predicting DNA content and mitotic phases. To investigate which classes of features were most useful for classifying DNA content and mitotic phases, we systematically excluded each of the feature classes from our analysis (the baseline analysis is shown as "none") and examined the effect of doing so on the results. Note that omitting any particular class of morphological features has very little impact on the overall accuracy of the approach, likely because many features are correlated and these phenotypes can be detected using many different features. Interestingly, performing the analysis using only the darkfield features (that is, leaving out all brightfield features, second row from the bottom) does substantially reduce the accuracy whereas using only brightfield features (that is, leaving out all darkfield features, last row) only slightly reduces accuracy. Thus, the brightfield features are much more informative then the darkfield features for these phenotypes.

Predicted cell cycle phases

Actual cell cycle phases

	G1/S/G2	Prophase	others	Actual fraction of population
G1/S/G2	85.83	9.17	5.01	88.65
Prophase	28.37	67.57	4.05	11.07
others	0	0	100	0.27

Supplementary Table 4 | Detailed results of cell cycle phase prediction after mitotic phase block. This display indicates the types of errors made by the system (i.e. the confusion matrix of the classification). For example, 88.65% of all cells in the sample were in G1/S/G2. 85.83% of these were correctly classified (predicted) by the machine learning system as in G1/S/G2. After treatment with 50µM Nocodazole, a prophase blocking agent the fraction of cells in prophase is increased to 11.07% as compared to 1.88% for the untreated cells (see Supplementary Table 2).. The increase in prophase was confirmed by visual inspection using the IDEAS software and provides evidence for the effectiveness of the phase blocking agent.

Predicted cell cycle phases

Actual cell cycle phases

	G1	S	G2	M		Fraction of population
G1	70.24	14.69	4.91	10.16		7.84
S	6.21	90.13	1.76	1.89		18.45
G2	0.63	0.51	96.78	2.07		71.75
M	20.35	4.90	30.71	44.04		1.96

Supplementary Table 5 | Detailed results of cell cycle phase prediction for fission yeast cells. This display indicates the types of errors made by the classifier (i.e. the confusion matrix). For example, 71.75% of all cells in the sample were in G2. 96.78% of these were correctly classified (predicted) by the machine learning system as in G2. Of the ~3.22% incorrectly classified, most were miss-classified as M (2.07%) followed by G1 (0.63%) and S (0.51%).

Reshaped image size	Correlation in prediction of DNA content
30x30 pixel	0.524±0.072
35x35 pixel	0.744±0.019
40x40 pixel	0.892±0.009
45x45 pixel	0.899±0.009
50x50 pixel	0.900±0.007
55x55 pixel	0.896±0.007
60x60 pixel	0.896±0.008

Supplementary Table 6 | Impact of image size on machine learning predictions. Reshaping the image size does not influence the results of the machine learning algorithm. We repeated our analysis where we reshaped the images to different sizes. The method is robust to the image size, as long as no parts of the cells are cropped (typical cell size: ~30-35x30-35 pixels).

Supplementary Notes

SUPPLEMENTARY NOTE 1 | Protocol for the analysis pipeline

Please note that an updated version of this tutorial is available at:
www.cellprofiler.org/imagingflowcytometry

STEP 1: EXTRACT SINGLE CELL IMAGES AND IDENTIFY CELL POPULATIONS OF INTEREST WITH IDEAS *SOFTWARE*

a. Open the IDEAS analysis tool (we used version 6.0.129), which is provided with the ImageStreamX instrument.
b. Load the .rif file that contains the data from the imaging flow cytometer experiment into IDEAS using File > Open. Note that any compensation between the fluorescence channels can be carried out at this point. The IDEAS analysis tool will generate a .cif data file and a .daf data analysis file (we provide a data file on www.cellprofiler.org/imagingflowcytometry).
c. Perform your analysis within the IDEAS analysis tool following the instructions of the software and identify cells that have each phenotype of interest, using a stain that marks each population. This is known as preparing the "ground truth" (expected result) annotations for the phenotype(s) of interest. In cases when a stain has been used to mark the phenotype(s) of interest in one of the samples, any parameters measured by IDEAS can be used to assign cells to particular classes. In the example data set, the PI (Ch4) images of pH3 (Ch5) positive cells (**Supplementary Figure 2**) are used to identify cells in various mitotic phases. An example IDEAS .daf file in which the settings for this are stored can be found on www.cellprofiler.prg/imagingflowcytometry.
d. Export the experiment's raw images from IDEAS in .tif format, using Tools > Export .tif images. In the opened window, select the population for which you want to export the images and select the channels you want to export. Change the settings Bit Depth to '16-bit (for analysis)' and Pixel Data to 'raw (for analysis)' and click OK. This will export images of the selected population into the folder where you placed your .daf and .cif files. In our example, we exported the cell's brightfield (Ch3), darkfield (Ch6) and PI (Ch4) images (the PI images are only needed to extract the ground truth of the cell's DNA content).

e. Move the exported .tif images into a new folder and rename it with the name of the exported cell population.
f. Repeat step d. and e. for all cell populations you are interested in (we exported Anaphase, G1, G2, Metaphase, Prophase, S and Telophase). You can find a zip-archive of the .tif images of the populations exported from the provided example data set on www.cellprofiler.org/imagingflowcytometry.

STEP 2: PREPROCESS THE SINGLE CELL IMAGES AND COMBINE THEM TO MONTAGES OF IMAGES USING MATLAB

To allow visual inspection and to reduce the number of .tif files, we tiled the images for the brightfield, darkfield and PI images to montages of 15x15 images. Both steps are implemented in Matlab and can be found in **Supplementary Code 1**. The provided Matlab function runs for the exported .tif images from step 1. To adjust the function for another data set, perform the following steps:

a. Open Matlab (we used version 8.0.0.783 (R2012b)).
b. Open the provided Matlab function (**Supplementary Code 1)** in the editor window by clicking 'Open' in the toolstrip.
c. Adjust the name of the input directory (folder) where the folders containing the single .tif images are located that were extracted from IDEAS in step 1 (in the example we used './Step2_input_single_tifs/').
d. Adjust the name of the output directory where the montages should be stored (in the example we used './Step2_output_tiled_tifs/').
e. Adjust the name of the folders where the single .tif images are located (in the example these are 'Anaphase', 'G1', 'G2', 'Metaphase', 'Prophase', 'S' and 'Telophase').
f. Adjust the name of the image channels as they were exported from IDEAS in step 1 (in the example we used 'Ch3' (brightfield), 'Ch6' (darkfield) and 'Ch4', PI stain).
g. Adjust the size of images (we have used 55X55 pixels for each image – this will depend on the size of the cells imaged and also the magnification).
h. Save the Matlab script by clicking 'Save' in the toolstrip.
i. Run the Matlab script by clicking 'Run' in the toolstrip and check that the montages appear in your designated output folder. The montages of 15x15 images that we

created from the example data set are provided on www.cellprofiler.org/imagingflowcytometry.

STEP 3: SEGMENT IMAGES AND EXTRACT FEATURES USING CELLPROFILER

To extract morphological features from the brightfield and darkfield images and to determine the ground truth DNA content we used the imaging software CellProfiler.

a. Open CellProfiler (we used version 2.1.1).
b. Load the provided CellProfiler project (**Supplementary Code 2**), including the pipeline within it, using File > Open Project.
c. Specify the images to be analyzed by dragging and dropping the folder where the image montages that were created in step 2 are located into the white area inside the CellProfiler window that is specified by 'File list'.
d. Click on 'NamesAndTypes' under the 'Input modules' and adjust the names of the image channels as they were exported from IDEAS and specified in step 2 f. Then click on Update.
e. Adjust the pipeline (which was loaded as part of Step b) if needed by adding or adjusting analysis modules (visit www.cellprofiler.org for tutorials on how to use CellProfiler). In the provided CellProfiler pipeline, we defined a grid that is centered at each of the 15x15 single cell images. We extracted features for the darkfield images (granularity, radial distribution, texture, intensity) based on the entire square image containing each cell. In other words, we did not attempt to measure darkfield properties within each individual cell by segmenting each cell, because the darkfield image is recorded at a 90° angle to the brightfield image and thus does not align with it. Further, darkfield does not necessarily depict the physical shape of the cell as is the case for brightfield. Next, we segmented the brightfield images (that is, identified individual cell borders) without using any stains, but by smoothing the images (CellProfiler module 'Smooth' with a Gaussian Filter) followed by edge detection (CellProfiler module 'EnhanceEdges' with Sobel edge-finding) and by applying a threshold (CellProfiler module 'ApplyThreshold' with the MCT thresholding method and binary output). We close the obtained objects (CellProfiler module 'Morph' with the 'close' operation) and use them to identify the cells on the grid sites (CellProfiler module 'IdentifyPrimaryObjects'). To filter out secondary objects (such as debris), which are typically smaller than the cells, on the single cell images we measure the sizes of secondary objects (if there are any) and neglect the smaller

objects. Then we extract features for the segmented brightfield images (granularity, radial distribution, texture, intensity, area and shape and Zernike polynomials). In a last step, we extract the intensity of the PI images that we use as ground truth for the DNA content of the cells. The complete CellProfiler pipeline with the parameters used in our analysis can be found in **Supplementary Code 2**.

f. Specify the output folder by clicking on 'View output settings' and selecting an appropriate 'Default Output Folder'.
g. Extract the features of the images by clicking on 'Analyze Images'. The extracted features from the brightfield and darkfield images as well as the intensity of the PI images in .txt-format are provided on www.cellprofiler.org/imagingflowcytometry.

STEP 4: MACHINE LEARNING FOR LABEL-FREE PREDICTION OF THE DNA CONTENT AND THE CELL CYCLE PHASE OF THE CELLS

I. Data preparation

a) Open Matlab (we used version 8.0.0.783 (R2012b)).
b) Open the provided Matlab function (**Supplementary Code 3**) in the editor window by clicking 'Open' in the toolstrip.
c) Adjust the name of the input directory where the folders containing the features in .txt format are located that were extracted from CellProfiler in step 3 (in the example we used './Step3_output_features_txt/').
d) Adjust the name of the output directory where the montages should be stored (in the example we used the current working directory).
e) Adjust the name of the feature .txt files of the different image channels as they were exported from CellProfiler (in the example these are 'BF_cells_on_grid.txt' for the brightfield features, 'SSC.txt' for the darkfield features, 'Nuclei.txt' for the DNA stain that we used as ground truth for the machine learning).
f) Change the name of the cell population/classes you extracted, provide class labels for them and specify the number of montages you created in step 2 for each of the cell populations/classes.
g) Specify the number of grid places that are on one montage as specified in step 2 (in our example we used 15x15=225).
h) Specify which features exported from CellProfiler in step 3 should be excluded from the subsequent analysis. Features that should be excluded are those that relate to the cells' positions on the grid. For the darkfield images we also excluded features

that are related to the area of the image, since we did not segment the darkfield images (the features we used for subsequent analysis can be found in **Supplementary Table 1**).

i) Save the Matlab function by clicking 'Save' in the toolstrip.
j) Run the Matlab function by clicking 'Run' in the toolstrip. The Matlab function excludes data rows with missing values corresponding, e.g., to cells where the segmentation failed or to grid sites that were empty. It combines the brightfield and darkfield features to a single data matrix and standardizes it (Matlab function 'zscore') to render all features to the same scale. Finally the feature data of the brightfield and darkfield images as well as the ground truth for the DNA content and the cell cycle phases are saved in .mat format (you can find the resulting data on www.cellprofiler.org/imagingflowcytometry).

II. LSboosting for predicting DNA content

We predict the DNA content of a cell based on brightfield and darkfield features only. This corresponds to a regression for which we used least squares boosting as implemented in the Matlab function 'fitensemble' under the option 'LSBoost'.

a) Open Matlab (we used version 8.0.0.783 (R2012b)).
b) Open the provided Matlab function (**Supplementary Code 4**) in the editor window by clicking 'Open' in the toolstrip.
c) Adjust the name of the input data containing the features that was created in step 4 I. to be used for regression.
d) Adjust the name of the ground truth data for the DNA content that was created in step 4.I. to be used to train the regression.
e) Save the Matlab function by clicking 'Save' in the toolstrip.
f) Run the Matlab function by clicking 'Run' in the toolstrip. In our example we used the settings 'LearnRate' equal to 0.1 and used standard decision trees 'Tree' as the weak learning structure. To fix the stopping criterion (corresponding to the amount of weak learners that is used to fit the data) we performed internal cross-validation (see below). The data is split into a training set (consisting of 90% of the cells) and a testing set (10% of the cells). Then the algorithm is trained on the training set for which the ground truth DNA content of the cells is provided, before it is used to predict the DNA content of the cells in the test set without providing their ground

truth DNA content. The predicted DNA content is provided on www.cellprofiler.org/imagingflowcytometry.

III. RUSboosting for predicting mitotic cell cycle phases

We predict the mitotic cell cycle phase of a cell based on brightfield and darkfield features only. This corresponds to a classification problem for which we used the boosting with random undersampling implemented in the Matlab function 'fitensemble' under the option 'RUSBoost'.

a) Open Matlab (we used version 8.0.0.783 (R2012b)).
b) Open the provided Matlab function (**Supplementary Code 5**) in the editor window by clicking 'Open' in the toolstrip.
c) Adjust the name of the input data containing the features that was created in step 4 I. to be used for regression.
d) Adjust the name of the ground truth data for the phases that was created in step 4.I. to be used to train the regression.
e) Save the Matlab function by clicking 'Save' in the toolstrip.
f) Run the Matlab function. In our example we used the settings 'LearnRate' equal to 0.1 and specified the decision tree structure that we used as the weak learning structure by setting the number of leafs 'minleaf' to 5. To fix the stopping criterion (corresponding to the amount of weak learners that is used to fit the data) we performed internal cross-validation (see below). Again, the data is split into a training set (90% of the cells) and a testing set (10% of the cells). Then the algorithm is trained on the training set for which the ground truth cell cycle phases of the cells is provided, before it is used to predict the cell cycle phase of the cells in the test set without providing their ground truth cell cycle phases. To show that the label-free prediction of cell cycle phases is robust we performed a ten-fold cross-validation. The predicted cell cycle phases are provided on www.cellprofiler.org/imagingflowcytometry.

Internal cross validation to determine the stopping criterion

To prevent overfitting the data and to fix the stopping criterion for the applied boosting algorithms, we performed a five-fold internal cross-validation. To this end, we split up the training set into an internal-training (consisting of 80% of the cells in the training set) and

an internal-validation (20% of the cells in the training set) set. We trained the algorithm on the internal-training set with up to 6,000 decision trees. We then predicted the DNA content/cell cycle phase of the inner-validation set and evaluated the quality of the prediction as a function of the used amount of decision trees. The optimal amount of decision trees is chosen as the one for which the quality of the prediction is best. We repeat this procedure five times and determine the stopping criterion for the whole training set as the average of the five values for the stopping criterion obtained in the internal cross-validation.
